# Seasonal influence on sperm parameters, scrotal measurements, and serum testosterone in Ouled Djellal breed rams in Algeria

**DOI:** 10.14202/vetworld.2017.1486-1492

**Published:** 2017-12-20

**Authors:** S. Belkadi, B. Safsaf, N. Heleili, M. Tlidjane, L. Belkacem, Y. Oucheriah

**Affiliations:** Department of Veterinary Science, Laboratory ESPA, Veterinary and Agricultural Sciences Institute, Hadj Lakhdar University, Batna - 05000, Algeria

**Keywords:** scrotal circumference, season, semi-arid area, spermatozoon, testosterone, weight

## Abstract

**Aim:**

This study was conducted to determine the effect of seasonal variations on testosterone serum concentration, body weight, scrotal circumference, and some sperm parameters in rams living in a semi-arid region of eastern part of Algeria.

**Materials and Methods:**

Blood samples were taken monthly from eight Ouled Djellal rams, aged between 3 and 4 years, in the Technical Institute of Breeding “ITELV” located at Ain M’lila City. Sperm were collected by an electro-ejaculator once a month for 1 year (spring, summer, autumn, and winter: 3 times/season).

**Results:**

Mean values of volume, mass motility, live sperm, and scrotal circumference were higher during spring (p<0.05) with 1.23±0.26 mL, 3.39±1.07, 79.16±15.82%, and 36.29±1.91 cm, respectively; whereas, the sperm concentration was higher during autumn with 1.19±0.56×10^9^ spz/ml compared to 0.46±0.13×10^9^ spz/mL to spring. The season influenced significantly the percentage of abnormal sperm (p<0.05), especially during winter (6.47±2.12%), but had no influence on the weight of rams. Seasonal hormonal activity was high with 4.89±2.06 ng/mL and 3.09±1.35 ng/mL of testosterone in mating seasons (spring and autumn, respectively), knowing that the sexual season is not marked too much in these latitude.

**Conclusion:**

We can conclude that testosterone concentration is strongly correlated with the scrotal circumference and that the season has a significant influence on spermatic parameters, and that despite the large variations in sperm production, the rams can be used throughout the year.

## Introduction

Sheep farming in Algeria constitutes 50% of the agricultural gross domestic product and the sheep number has increased from 17.5 to 26.6 million head, with an average annual increase of 4.4% over the 10-year period (2003-2013) [1]. The sheep raising is concentrated in the steppe and constitutes a large animal resource of the country. This breed tends to dominate other blood, improving its fertility to increase livestock productivity and reproductive efficiency [2]. The mutton meat is the most favorable red meat for consumption and is the favorite in religious and traditional festivals.

Eight major breeds have been identified so far. Some of these breeds show strong adaptation capabilities to harsh environmental conditions (such as water and/or food scarcity and high temperatures). Among them, one breed, the Ouled Djellal (OD) also known as the great white Arabian breed, bred in the arid and semi-arid regions and subjected to clear preference of the farmers [3]. The race OD supplants and jeopardizes the existence of other ovine Algerian breeds due to its high zootechnical potential [4]. Moreover, that obvious prevalence (58% of the Algerian sheep) on the economical market induces a very sensitive situation for the other breeds, especially because some of them are submitted to uncontrolled crossbreeding with the favored breed and/or to real marginalization [5].

Ram’s sexual behavior can be influenced by many factors, including season of the year, genetics, breed differences, hormonal influence, post-weaning management, temperature, and nutrition. However, the photoperiod is the main environmental factor affecting sheep reproduction [6]. Thus, in subtropical areas, many goat and sheep breeds express seasonal variation of their sexual activity. This is similar to that observed in temperate zones, except in terms of duration of sexual activity expression due to the amplitude of photoperiod variation [7]. During ram’s life, body weight, scrotal circumference, and testosterone levels change under the influence of several internal and external factors. Data on ram’s reproduction reveal a complex relationship between the development of the neuroendocrine system, the concentration of testosterone, the development of certain parts of body, and sexual maturation [8]. Therefore, knowledge of the quality of sperm can influence reproductive factors in order to improve genetically the breed and increase the numerical productivity of the herd [9]. Seasonal variation in mammals’ breeding is an adaptation to the annual environmental changes [10]; therefore, they are an important factor influencing the quantity and quality of sperm. Thus, in temperate regions, sheep have a clear seasonal sexual activity; where males exhibit changes in behavior, testicular weight, and qualitative and quantitative sperm production coinciding with decreased day length [11]. Testosterone plays a central role in the control of spermatogenesis from the testicular stage (differentiation of spermatogonia to spermatids) and decreases germ cell apoptosis [12], in the development and maintenance of sexual behavior in rams [13]. Blood levels vary according to race, age, nutrition, and season [14].

The present study aimed to evaluate the influence of the season on spermatozoa and blood testosterone concentration in OD rams in semi-arid zones. In order to select the best spawners at our insemination centers during or outside the mating season, it is useful to have specific and objective information about testicular development, sperm characteristics, and hormonal status.

## Materials and Methods

### Ethical approval

The blood samples were collected from the animals for serum extraction. The ethical considerations in accordance to the Institute Animal Ethics Committee related to animal handling were observed to ensure no pain to animal during sampling.

### Experimental location and climate

The study was performed at ITELV-Ain M’lila (Technical Institute of Breeding), a semi-arid region in north steppic region of eastern part of Algeria at 775 m of altitude, 36°2’13” North of latitude and 6°34’33” East of longitude. Each season lasts 3 months. The area is characterized by very cold winters with a minimum between 1°C and 5°C in January and very hot and dry summers with temperatures between 33°C and 40°C recorded in August.

### Animals

Eight white OD rams, aged between 3 and 4 years, were used at the Technical Institute of Breeding (ITELV). At the center, the ram effect is the practice used for reproduction. Two months before the fight, these rams are removed from the herd and reintroduced at both breeding periods. Two days before harvesting of blood samples and sperm collection, the rams are removed from the herd.

These rams were dewormed once a year and received a diet consisting of barley straw or wheat, alfalfa hay, and concentrate. The water was *ad-libitum*. The mean score of the rams’ body condition varied between 2.5 and 3.0 (scale=0-5) according to Dedieu *et al’s*. method [15]. The mating is carried out in two periods, one in spring (April-May) and the other in autumn (October-November).

### Blood samples for hormonal dosage

Blood samples were taken monthly from the jugular vein of each ram for 1 year. The blood was immediately centrifuged and the serum obtained was frozen at −20°C until testosterone was assayed. The serum testosterone concentration was determined using Enzyme Immunoassay kits (ST AIA – PACK Testosterone for quantitative measurement of testosterone in serum on TOSOH AIA System Analyzers).

### Weight and testicular weight measurement

A weighing balance scale was used for weighing and a zootechnical tape measure was used for measuring scrotal circumference at the largest diameter of the scrotum.

### Sperm collection and evaluation

Semen was collected with an electro-ejaculator from all the rams. The volume was read by means of a graduated tube; the concentration of 10^9^/ml was determined by counting on a Malassez slide and the mass motility was subjectively evaluated by examining an undiluted and colored sperm drop under a microscope equipped with a heating platform. Motility was assessed at magnification 10× to assess the intensity of the waves formed by sperm movements; a score ranging from 0 to 5 was attributed [16]. However, the vitality was assessed by counting after Eosin-Nigrosin staining, to determine the percentage of live (Eosin negative) spermatozoa.

### Statistical analysis

The results are presented with standard deviation. The analysis of the ANOVA variance was used to determine seasonal variations in sperm parameters and testosteronemia, and measurements (weight and testicular) were performed using Graph Pad Prism 5.03. The differences detected were considered significant when p<0.05.

## Results

### Seasonal variations in body weight and scrotal measurements

#### Weight

Table-1 shows that weight did not vary significantly (>0.05) with maximum averages during spring (94.71±3.79 kg) and minimal during autumn (91.96±4.10). Although we observe a little decrease, there was no significant change during the winter and summer (93.46±5.82 kg and 92.45±2.79 kg, respectively) (Table-1).

**Table-1:** Seasonal variations in body weight, scrotal circumference, and serum testosterone. Pearson’s correlation between scrotal circumference and testosterone.

Periods	Weight (kg)	Scrotal circumference (cm)	Testosterone (ng/mL)
Winter (n=24)	93.5±5.8	34.0±2.1	2.42±1.59
Spring (n=24)	94.7±3.8	36.3±1.9	4.89±2.06
Summer (n=24)	92.5±2.8	33.3±1.9	2.21±1.66
Autumn (n=24)	92.0±4.1	34.9±2.1	3.09±1.35
Statistical significance	NS	a*	a*; b*

^a^Spring versus summer; ^b^Winter versus spring;

* p<0.05

### Scrotal circumference

Seasonal influence on scrotal circumference was statistically significant (p<0.05). The highest averages were recorded during spring (36.3±1.9 cm) and in autumn during breeding months, while the lowest averages were observed during summer (33.3±1.9 cm) (Table-1).

### Seasonal variations of serum testosterone

Statistical analysis showed that testosterone serum levels were significantly affected by breeding months. Thus, we observed a significant difference (p<0.05) compared to spring versus summer and winter versus spring. As for seasonal averages, the highest values were observed in spring and autumn with 4.89±2.06 ng/mL and 3.09±1.35 ng/mL, respectively, coinciding with the periods of maximum sexual activity. However, there was no correlation between testosterone levels and spermatic parameters (Table-1).

### Seasonal variations of spermatic parameters

#### Volume

Season influenced significantly the volume of collected sperm (p<0.05); sperm production was maximal during spring (1.23±0.26 mL), while the lowest volume was obtained in winter (0.94±0.11) (Table-2).

**Table-2:** Seasonal variations of spermatic parameters (volume, massal motility, sperm concentration, live and abnormal spermatozoa).

Periods	Volume (mL)	Massal motility	Sperm concentration (×10^9^/mL)	Live spermatozoa (%)	Abnormal spermatozoa (%)
Winter (n=23)	0.94±0.11	2.62±1.00	0.59±0.25	74.4±10.1	6.47±2.12
Spring (n=24)	1.23±0.26	3.39±1.07	0.46±0.13	79.2±15.8	3.71±1.71
Summer (n=24)	1.14±0.20	2.16±0.84	0.54±0.26	58.3±14.9	2.47±0.88
Autumn (n=24)	1.09±0.18	2.83±1.33	0.96±0.27	80.0±9.03	4.04±1.83
Statistical significance	b*		c**; d***; e*	a*; c*	b*; f**

^a^Spring versus summer; ^b^Winter versus spring; ^c^Summer versus autumn; ^d^Spring versus autumn; ^e^Winter versus autumn; ^f^Winter versus summer.

* p<0.05;

** p<0.01;

*** p<0.001

#### Massal motility

Results indicated that massal motility was not significantly (p>0.05) affected by season (Table-2). However, the higher average value was observed in spring (3.39±1.07), which decreased in summer, and then reached a new peak in autumn (2.83±1.33) and decreased again during winter.

#### Sperm concentration

The mean averages showed significant difference (p<0.001), with a greater sperm concentration in autumn (1.19±0.56×10^9^ spz/mL) and low concentration (0.46±0.13×10^9^ spz/mL) in spring (Table-2).

#### Live spermatozoa

Results obtained showed a significant influence of season on the percentage of live spermatozoa (p<0.05). The maximum averages (79.16±15.82) were observed during spring, which corresponds to the mating period, while lowest was noted during summer.

#### Abnormal sperm

Monthly average results of the anomaly rate showed a rise from the end of autumn to the end of winter with significant rate during the winter (6.47±2.12%) followed by a remarkable decrease with a minimal average recorded during the summer (2.47±0.88%) (Table-2).

## Discussion

Several studies have demonstrated that season has an influence on ram’s reproductive characteristics [17,18]. They reported that the standard method of evaluating the fertility of male breeding is the examination of sperm production [19]. We observed no significant difference (p>0.05) and no seasonal effect on the ram weight; this result could be explained by the fact that these rams are adults and raised under good conditions at the breeding institute. Kafi *et al*. [17] and Kridli *et al*. [6] reported that body weight was not affected by seasonal variation on Persian karakul rams while Avdi *et al*. [20] and Boucif *et al*. [21] observed seasonal changes.

For scrotal circumference, Maksimovic *et al*. [8], Abba *et al*. [22], and Ghorbankhani *et al*. [23] have shown that testicular size is often used to evaluate ram’s fertility and that testicular morphometry was considered as the predictor of sperm production. Similarly, Belkhiri *et al*. [24] reported that the measurement of scrotal circumference in OD rams could be used in breeding centers to select suitable breeding male for artificial breeding purpose. Thus, the highest measurements observed in spring and autumn season coincide with the periods of breeding practiced in this farm, where the increase in testicular volume is associated with an increase of sperm production. Authors reported also that, during the resumption of sexual activity season, there is a time lag in the resumption of this activity, which begins a month to a month and a half earlier in males than in females [25]. Our results are almost equivalent to those reported by Allaoui *et al*. [26] on adult rams. The lowest average was observed in summer, conversely to the observation of Boucif *et al*. [21] during winter in western part of Algeria over rams OD. On Lacune rams at Brazil [27] a significant difference was found (p<0.05) between winter (31.0±3.4 cm) and spring (34.1±2.5 cm), and no significant difference between the other seasons, which is consistent with our results. The peak of measurements observed in September is in agreement with the observations of Ghorbankhani *et al*. [23] in Iran in Sinjabi rams and Kafi *et al*. [17] in Persian Karakul rams.

The observed morphometric variations correlated with serum testosterone levels (Table-3), which follow the same seasonal pattern. Since testosterone is the hormone that controls testicular, epididymal, and accessory glands’ activities, several authors argue that testosteronemia is a good marker of quantitative and qualitative production of sheep sperm [28-31]. Darbeïda *et al*. [32], who worked on OD rams, found that testosterone reached a peak in summer and a low rate in winter; while Loubser *et al*. [33] found that the peak of testosterone blood concentration (10.9 ng/mL) in the Angora breed reached at the beginning of March. Our results seem to be in agreement with those of Benia *et al*. [34] who found that testosterone concentrations are maximal during spring and autumn months, and that OD rams showed continuous spermatogenetic activity during all seasons. In addition, serum testosterone levels were low during July where the temperature was very high on the day of collection. This is in agreement with the results obtained by Maurya *et al*. [35] in Malpura rams subjected to high temperatures (42°C and 55% of relative humidity) for 6 h per day during 10-16 h. It was also reported in bulls and boars that testosteronemia was found to decrease during high heat, but tends to recover if heat stress lasts more than 2 weeks [36].

**Table-3:** Average number of live spermatozoa and sperm doses usable for AI (400×10^6^spz live) obtained per ram per collection.

Periods	Number of live spermatozoa (millions) per collection	Sperm doses usable for AI per collection
Winter (n=24)	413±154	1.03±0.38
Spring (n=24)	448±140	1.12±0.35
Summer (n=24)	359±215	0.90±0.54
Autumn (n=24)	837±325	2.09±0.81
Statistical significance	a***; b**; c**	a***; b**; c**

^a^winter versus autumn, ^b^spring versus autumn, ^c^summer versus autumn.

** p<0.01;

*** p<0.001. AI=Artificial insemination

The values recorded for the ejaculated volume were lower in winter as reported by Boucif *et al*. [21] and Pourseif *et al*. [37]. In addition, an increase of volume was noted at the end of June to the end of September and a decrease toward the end of October. This decrease is being maintained until the end of February to increase gradually and reach a new peak in March (1.35±0.15 mL). It was lower than that of 1.99 mL recorded using artificial vagina by Aissaoui *et al*. [38]. The increase in sperm volume from the end of winter to its peak in spring is in agreement with Kafi *et al’s*. [17] results. On the other hand, Aissaoui *et al*. [38] showed that the quantity of ejaculated semen reaches maximum values in June and seems to evolve with the daily illumination duration.

The fluctuations of mass motility were not really affected by the photoperiod as shown by Colas [39] and Aller *et al*. [40]. We note that motility follows the same trend as that of testosterone. However, it seems to be affected by the high temperatures because we noted very low values in July (where the ambient temperature reached 47°C), which is in agreement with the finding of Ghozlane *et al*. [41]. Kafi *et al*. [17] have demonstrated in a study on Iran Karakul breed rams that high temperatures do not affect mass motility of semen and have concluded that higher sperm quality is obtained toward the end of summer.

Sperm concentration is the most variable parameter (more than double in spring to autumn). Our results are comparable to those found by Deldar *et al*. [42], Zamiri *et* *al*. [18], and Moghaddam *et al*. [43], with maximum values in autumn and decreased ones in winter. Similar observations have been reported in the Ile-de-France breed, where Colas [44] noted that sperm-fertilizing power is significantly lower in spring (March-April) than in autumn (September) in the adult ram.

Percentage of live sperm is significantly affected by the season, the highest mean value is recorded in autumn; while the lowest value is in summer. It should be noted that Pourseif *et al*. [37] observed the highest rate of sperm vitality from September to December, thus meeting the observations of Dufour *et al*. [45] and Moghaddam *et al*. [43], with higher values in autumn (October-November) than in spring. However, Kafi *et al*. [14] observed the highest rate in September (97.6±13.1%) and the lowest one in January (82.1±11.3%). Aller *et al*. [40] found no significant effect of season on live spermatozoa.

A significant effect of season on the percentage of abnormal spermatozoa, especially in winter, is noted, in comparison with low values recorded in spring and summer (p<0.05), which is in agreement with results obtained by Mandiki *et al*. [46]. The increased rate of sperm abnormalities can be explained by decreased serum testosterone concentration, reduced seminiferous tube diameter and spermatogenetic activity, or a maturation defect at the epididymal level [47]. The best indicator of seasonal effects on sperm quality is sperm abnormality, which is generally proportional to the fertility test [39]. Zamiri *et al*. [18] found that the rate of sperm abnormalities increased from January to March, with rates ranging from 7.5% to 14.2%. The average morphological abnormalities of spermatozoa recorded during our experiment were <15%, percentage above which sperm is considered to be of poor quality [13].

Finally, the mean number of live spermatozoa per collection and thus the theoretical mean number of semen doses usable for artificial insemination (AI) obtained per male varied from simple to double between winter and autumn (Table-4).

**Table-4:** Pearson’s correlation between testosterone, scrotal circumference, and sperm parameters.

Testosterone	Testosterone (ng/mL)	Scrotal circumference (cm)	Weight (kg)	Volume (mL)	Massal motility	Sperm concentration (×10^9^/ml)	Live spermatozoa (%)	Abnormal spermatozoa (%)
Scrotal circumference (cm)	0.974							
Weight (kg)	0.724	0.639						
Volume (mL)	0.690	0.563	0.311					
Massal motility	0.943	0.986	0.684	0.428				
Sperm concentration (×10^9^/ml)	−0.249	−0.062	−0.753	−0.280	−0.071			
Live spermatozoa (%)	0.647	0.798	0.345	−0.001	0.856	0.357		
Abnormal spermatozoa (%)	−0.143	−0.007	0.217	−0.811	0.155	0.086	0.471	

## Conclusion

The observed changes on sperm parameters during the period of reproduction indicated a relationship between serum testosterone massal motility and scrotal circumference. The best sperm was obtained during spring and autumn, coinciding with usual matting periods in our farms. Moreover, the average number of doses usable for AI tends to be higher in autumn and lower in summer and winter. Overall, our results illustrate that seasonal variations of sperm concentration do not prevent using OD breed rams throughout the year for collection of sperm to be used for AI.

## Authors’ Contributions

SB, BS, and NH designed the experiment protocol. SB and LB carried out the experiment work. SB, BS, NH, and MT were involved in data analysis and scientific discussion. SB, BS, NH, MT, LB, and YO drafted and revised the paper. All authors read and approved the final manuscript.
